# Health-related impact on quality of life and coping strategies for chikungunya: A qualitative study in Curaçao

**DOI:** 10.1371/journal.pntd.0005987

**Published:** 2017-10-09

**Authors:** Jelte Elsinga, Martin P. Grobusch, Adriana Tami, Izzy Gerstenbluth, Ajay Bailey

**Affiliations:** 1 University of Groningen, University Medical Center Groningen, Department of Medical Microbiology, Groningen, The Netherlands; 2 Center of Tropical Medicine and Travel Medicine, Department of Infectious Diseases, Academic Medical Center, University of Amsterdam, Amsterdam, Netherlands; 3 Curaçao Biomedical & Health Research Institute, Department of Epidemiology, Willemstad, Curaçao; 4 Epidemiology and Research Unit, Medical and Public Health Service of Curaçao, Willemstad, Curaçao; 5 International Development Studies, Department of Human Geography and Spatial Planning, Utrecht University, Utrecht, Netherlands; 6 Dr. T. M. A. Pai Endowed Chair in Qualitative Methods, Manipal University, Manipal, India; London School of Hygiene and Tropical Medicine, UNITED KINGDOM

## Abstract

**Introduction:**

Chikungunya is an emerging public health problem in tropical and subtropical regions, due to ongoing transmission and its incapacitating acute disease phase, and chronic sequelae. The disease is responsible for a major impact on Health Related Quality of Life (HRQoL), which may last several years. To our knowledge, this study is the first qualitative examination of HRQoL and coping strategies of chikungunya-infected individuals.

**Methods:**

Qualitative research methods consisted of 20 in-depth interviews and seven Focus Group Discussions (FGDs), n = 50. Analysis was based on the principles of the grounded theory.

**Results:**

Different impacts on HRQoL were reported. The physical and emotional domains of the HRQoL were mainly affected by chikungunya, while social and individual financial consequences were limited. Individual financial impact was limited through the universal health care program of Curaçao. Long-term lingering musculoskeletal and other manifestations caused significant pain and limited mobility. Hence, participants experienced dependency, impairment of normal daily life activities, moodiness, hopelessness, a change of identity, and insecurity about their future. The unpredictable nature and consequences of chikungunya gave rise to various coping strategies. Problem-focused coping styles led to higher uptake of medical care and were linked to more negative impact of HRQoL, whereas emotional coping strategies focusing on acceptance of the situation were linked to less uptake of medical care and more positive impact on HRQoL.

**Conclusions:**

This study provides an in-depth understanding of acute and long-term HRQoL impact of chikungunya. The results can better inform health promotion policies and interventions. Messages to the public should focus on promoting healthy and efficient coping strategies, in order to prevent additional stress in affected individuals.

## Author summary

Chikungunya is a disease caused by a virus, which is transmitted by mosquitoes. During the past years, major outbreaks of chikungunya have occurred in the Americas. Normally, chikungunya presents with an acute, fever-like disease. After acute disease, many people develop chronic disease manifestations, which are mainly characterized by joint pain. In this study, we examined the impact of chikungunya on quality of life and how people coped with these consequences. The results of this study show that people infected with chikungunya reported long-term pain and limitation of mobility. Consequently, impairment of normal daily life activities and several emotions were experienced; for example dependency, moodiness, and insecurity about their livelihood and their future. People narrated visiting a doctor and using treatment to cope with these consequences. However, the participants who accepted their chronic ‘diseased’ condition could better cope with the consequences of chikungunya than the participants who could not accept their chronic condition. The latter group kept searching for new treatments and frequently consulted doctors. It is important that doctors and health authorities are aware of these results, because this will aid them to improve care and promote better coping strategies of people affected by chikungunya.

## Introduction

Chikungunya is an arboviral disease mainly transmitted by the mosquito species *Aedes aegypti* and *Aedes albopictus* [1]. Chikungunya has (re-)emerged in the Americas in 2013 [2], when it rapidly spread from the islands of the Caribbean to the Latin-American mainland [2–4]. Since then, the Pan American Health Organization reported an approximated one million chikungunya cases per annum in the Americas [5]. However, during this introduction of chikungunya virus (CHIKV) in the Americas, more people might have been infected, since studies describing attack rates have reported that 35–90% of the population in these regions were infected by CHIKV [6,7].

Curaçao witnessed a chikungunya outbreak in 2014–2015, which culminated in October and November 2014. The epidemic ended early in 2015, when up to approximately 50,000–75,000 (attack rate: 33–50%) individuals had been infected in Curaçao [6].

Chikungunya typically consists of an acute phase and a (sub-)chronic phase [8]. The acute phase commonly presents with a sudden onset of fever and severe musculoskeletal pain lasting up to twelve days [8]. Other manifestations during acute disease may include, rash, fatigue, joint swelling and nausea [8,9]. Acute disease signs and symptoms eventually subside, but in the majority of cases linger on for months or years. Persistent joint or musculoskeletal pain and weakness are characteristic in its chronic presentation, and may be accompanied by fatigue, loss of vitality and neurologic manifestations [10]. This chronic disease course has major impact on the quality of life (QoL) of affected people [10].

Health Related Quality of Life (HRQoL) describes QoL in its relation with health conditions. The World Health Organisation (WHO) defines health as ‘a state of complete physical, mental, and social well-being not merely the absence of disease…’ [11]. The latter description is widely considered as a definition of QoL [12]. Hence, HRQoL is a multidimensional concept typically referring to physical, emotional and social well-being and may often include a measure of socio-economic well-being [12–14].

Studies addressing the impact of chikungunya on the HRQoL of an individual have been performed using standardized questionnaires for HRQoL as the SF-36 questionnaires. Most of these studies associate chronic chikungunya with reduced HRQoL-scores on both physical and mental components [6,15–17]. The impact on HRQoL varies with severity of disease, showing in particular substantial drops in HRQoL when patients are more severely affected by chikungunya [6]. The decrease of HRQoL-scores in both physical and mental components is remarkable and alarming, given the emerging spread of CHIKV.

HRQoL is influenced by individual subjective perceptions, expectations and coping strategies towards the health condition. Individuals showing the same clinical presentation of disease may exhibit different coping strategies, which can either positively or negatively impact the HRQoL [18]. Lazarus’s model of coping describes that emotional (e.g. acceptance, positive reappraisal) and problem-focused (targeting the cause of stress, e.g. treatment, physician visit) coping strategies are interdependent responses towards perceived stress. Hence together, these coping strategies account for an overall coping response [19,20]. Previous research on malaria and HIV have revealed that both emotional and problem-focused coping strategies can be linked to HRQoL outcomes [21–23]. Generally, problem-focused coping strategies are considered to improve HRQoL. However, a shift towards emotional coping strategies might be more appropriate when there is no treatment available [24]. Therefore, with their objective to improve HRQoL, health interventions should be grounded in knowledge on coping strategies that are employed by the targeted study population for various diseases.

Previous studies on HRQoL of chikungunya have linked decreased HRQoL scores to chikungunya manifestations, but have not attempted to obtain in-depth understanding of the different concepts of HRQoL; nor have they assessed coping strategies influencing the HRQoL [6,15–17].

This study is part of a larger mixed methods project, which was set up to investigate how chikungungya impacts on HRQoL. This qualitative study was designed to obtain an in-depth understanding of this topic, while a simultaneously performed survey provided a representative quantitative assessment of the impact of chikungunya on HRQoL in Curaçao [6]. Therefore, the two main research questions of the study presented here are (a) how is the impact of chikungunya on HRQoL concepts (physical, emotional, social life and economic well-being) experienced by people in Curaçao? and (b) what coping strategies are employed by those affected by chikungunya to reduce HRQoL impact?

## Materials and methods

### Ethics statement

The study was approved by the Medical Ethical Board of the Sint Elisabeth Hospital Curaçao (METC SEHOS; reference number: 2015–002). All subjects consented in writing to study participation.

### Study site and population

Curaçao is an island in the Caribbean Sea, formerly part of the Dutch Antilles but since 2010 an autonomous country within the Kingdom of the Netherlands [25]. Curaçao is located close to the Venezuelan coast and consists of an area of 444 square kilometres. The approximately 150 thousand inhabitants live mainly in Willemstad, the capital of Curaçao. The GDP per capita is 22,600 dollar (2012), which renders Curaçao a comparably affluent Caribbean island. Willemstad covers a big part of the South-Eastern part of Curaçao and constitutes the most important economic area of the island [26]. Urbanisation and Curaçao’s semi-arid climate with a rainy season from October to December [27], provide favourable living conditions for *Aedes* spp. The population consists of an Afro-Caribbean majority and diverse minorities being Latin American, Dutch, Portuguese, French, Levantine and South- or East-Asian people [27]. Generally, all inhabitants of Curaçao are insured for most medical expenses through a state sponsored health insurance scheme (Social Insurance Bank), through their employer, or through a private health insurance. The health system consists of a network of general practitioners for primary care. For hospitalization and specialized care patients are referred to the Sint Elisabeth Hospital, which is the main hospital of the island.

### Study design and participants recruitment

This study was set up as an exploratory, qualitative study using in-depth interviews (IDIs) and focus group discussions (FGDs). The consolidated criteria for reporting qualitative studies (COREQ) [28] were followed and presented in S1 Table. FGDs and IDIs were performed simultaneously, which allowed us to use FGDs to verify and contextualise topics which had emerged in IDIs. On the other hand, themes arising in FGDs could be more profoundly understood in IDIs. Interviews were performed in June and July 2015 up to the point of data saturation, which was evaluated and agreed upon within the research group. A total of 75 people participated in this study (age range: 18–97), of which 50 participated in one of the seven FGDs (4–10 participants per FGD) and 20 in IDIs (Table 1). To understand the caretakers’ perspectives, we conducted key-informant interviews with family members of chikungunya patients (n = 5).

**Table 1:** Participants’ characteristics.

	# participants	# female	Age range
**Focus groups (n = 7)**			
Residents from the Netherlands	8	6	61–71
Local youth	4	2	19–24
Koraalspecht	10	10	55–97
Seru Fortuna	9	8	18–70
Rooi santu	8	4	51–80
Souax	7	4	34–72
Interviewers of the survey	4	3	64–67
**In-depth interviews (n = 20)**			
Participants with chikungunya	20	12	36–87
Family members	5	2	-

### Participant recruitment IDIs

Potential participants of the IDIs were contacted via key informants (general practitioners) and snowballing (performed by the research group). Eligible participants were then recruited by the research group via telephone. Inclusion criteria for IDIs were adults with either (1) a serologically confirmed CHIKV infection during the chikungunya epidemic of 2014–2015 based on a positive IgM/IgG, using an ELISA test or (2) a history of an acute disease with chronic musculoskeletal pain during the chikungunya epidemic of 2014–2015, plus a positive IgM/IgG performed on screening for inclusion in this study. Participants were selected from different socio-economic strata, age categories and genders. IDIs were performed to provide deeper insights on HRQoL concepts and coping strategies, the interplay of coping strategies and HRQoL.

### Participant recruitment FGDs

Seven FGDs were performed amongst adults (regardless of having (had) chikungunya or not), which were invited for the FGDs via volunteers of neighbourhood centres and key informants (of social groups). Individuals from various representative socio-economic groups (ranging from low, middle, and high socio-economic statuses) of Curaçao were included, i.e. 1) residents born in the Netherlands, 2) local youth, 3–6) people from the neighbourhoods Koraalspecht, Seru Fortuna, Rooi Santu and Souax. Local survey-investigators had additional contextual information, which they received during the simultaneously performed quantitative survey on HRQoL of chikungunya, which has been published elsewhere [6]. Hence, a FGD was conducted among the survey-investigators to cross-check information that we received from IDIs, surveys and FGDs.

### Data collection

**Fig 1:**
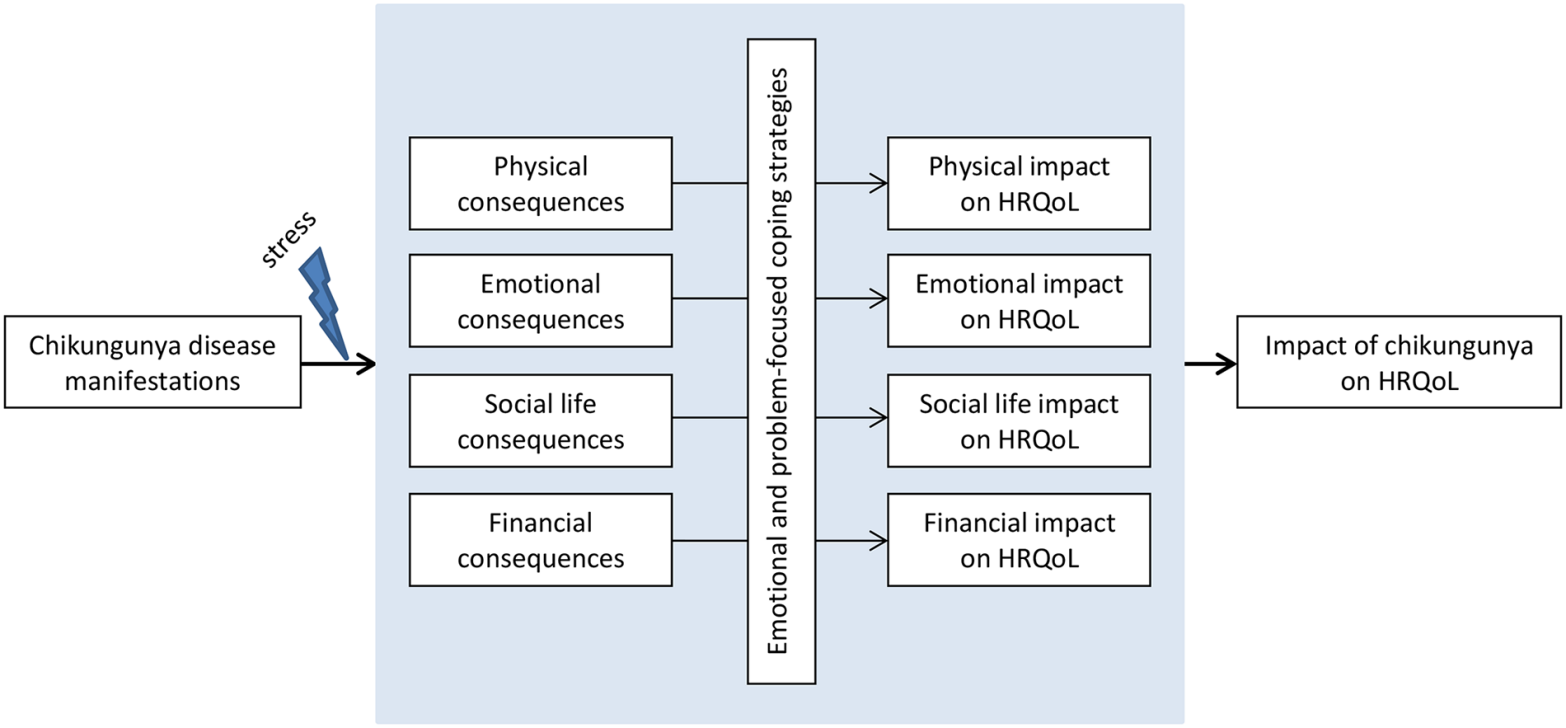
Theoretical framework.

Prior to data-collection study procedures and confidentiality were discussed, after which all participants consented in writing to study participation. Two local social workers with a wide experience with group discussions performed the FGDs and the IDIs, together with Jelte Elsinga. Interviews were conducted in neighbourhood centres, at the homes of participants, or in another place which was chosen by the participants. Only interviewers and participants were present during the IDIs and FGDs, which were held in Dutch, Papiamentu or Spanish. The concepts of the HRQoL (physical, emotional, social life and financial well-being) and coping strategies served as theoretical framework (Fig 1) for the interview and focus group guides (S1 and S2 Text). As reflected in the theoretical framework, we expected that chikungunya disease manifestations could induce stress to all concepts of the HRQoL. The ability to cope with the consequences of this stress determined the impact on HRQoL of an individual concept. The impact on all four concepts (i.e. physical, emotional, social life and financial well-being) of the HRQoL resulted in the final/overall impact on HRQoL of chikungunya (Fig 1). Interview and focus group guides were prepared and used as a guide in the FGDs and IDIs (S1 and S2 Text). These interview guides were evaluated and adapted after pilot interviews in Curaçao. The pilot interviews were included in the analyses of this study. Interviews were recorded, translated into Dutch and transcribed in verbatim. Field notes were made during and after interviews. When information started to repeat itself (data-saturation) the researcher discussed with the team the themes covered and decided to stop data collection.

### Data analysis

Data analysis was based on principles of the Grounded Theory [29], which helped to visualize the analyses and to move from data to theoretical explanations. Analyses were performed using Atlas.ti (version 7.5.4) software. Two cycles of inductive and/or deductive coding were employed. The first cycle of inductive and deductive coding resulted in 31 codes. In the second cycle, only deductive coding was employed. In the second cycle, the codes of the first cycle were categorized as per the concepts of the theoretical frame HRQoL and coping mechanisms, resulting in five (deductive) code families: (1) physical impact (codes: e.g. acute/chronic symptoms, lingering symptoms, daily life problems); (2) emotional impact (codes: e.g. emotions, dependency on others, confusion related to chikungunya symptoms); (3) impact on social life (codes: e.g. impact on social life, general impact of chikungunya, impact on society); (4) financial impact (codes: e.g. impact on work, insurance, financial costs of chikungunya); and (5) coping strategies (codes: e.g. doctor visit, treatment, (emotional) coping style).

## Results

The results of this study were structured in sections as per the concepts of the theoretical framework (Fig 1). The provided quotes serve to contextualize, illustrate or clarify the results of this study. Quotes are transliterations from the interview-recordings, and are presented without extensive editing to remain close to the cultural meanings that are in the data.

The epidemic of 2014–2015 in Curaçao was the first chikungunya outbreak on the island in recent times. Communities’ knowledge on chikungunya transmission routes and clinical presentation was therefore absent, or very limited at the onset of the chikungunya epidemic in Curaçao. During the epidemic, participants acquired knowledge of chikungunya from radio, television, newspapers, social media and social interactions (e.g. family, friends, neighbours). The key health communication messages came from the Ministry of Health of Curaçao, alternative health providers and personal experiences of the community. Due to the limited knowledge prior to the outbreak, these communications shaped participants’ knowledge regarding chikungunya, giving rise to varying perceptions on chikungunya sequelae and coping strategies.

### Physical impact of chikungunya

The community (as documented in FGDs) recognized chikungunya as being a debilitating disease with major acute and chronic impact on physical health. Musculoskeletal pains and stiffness were most prominently reported and perceived to cause physical impairment. However, the common symptoms that were described by the community included a wide range of symptoms for both the acute and chronic phase of chikungunya. Before onset of the disease, some individuals reported to have perceived a prodromal period in which they had ‘felt strange’ for days or weeks. Individuals, in the IDI, narrated how acute chikungunya had started with a sudden onset of symptoms with varying intensity. The reported symptoms included fever, swollen joints, musculoskeletal pains, weakness, stiffness, fainting, difficult breathing, headache, dizziness, change in sense of taste, itch, rash, incontinence, anorexia, numbness, tingling, diarrhoea and constipation. The sudden nature of acute chikungunya was reflected in situations where people could not get out of bed, a chair or car, or fell down when trying, thus increasing the risk of fall-related injuries.

Participant A: The way in which I realized it [that I was ill] was, I rose and stepped out of bed and I could not stand because my ankles gave away [failed].(…) So I got back in bed and I think ‘help! Am I paralysed or so?’ you know?

(Woman, IDI, aged 60–70 years)

Participants had different experiences with chikungunya symptoms per case, as described above. Some had not perceived long-term complaints. Others described a second ‘phase’ of disease, which lingered on, or started after acute symptoms had ceased. This second ‘phase’ referred mainly to the long-lasting complaints, typically characterized by musculoskeletal pain and weakness. Furthermore, additional chronic symptoms were reported, such as cramps, fatigue, ‘trigger fingers’ and numbness (paraesthesia). These long-lasting symptoms could be constant, but could also have a lingering nature, affecting different places alternatingly. According to some participants, their weak spots (for example old injuries) were the places where complaints of chikungunya particularly manifested.

Participant B: So, when I was young I do, I did ehm taekwondo and gymnastics too. And I had ehm, old pains from my wrists, ankle, tibia, shoulder. All the old pains had returned…

(Man, IDI, aged 30–40 years)

As was narrated by many individuals, the consequences of chikungunya resulted in challenging situations. On disease onset, when generally the most intense symptoms were perceived, people were commonly forced to stay in bed, sometimes without the ability to leave bed without help. The latter inconvenience was commonly described together with the difficulties participants faced in reaching the toilet. The activities of daily living were affected for almost all the participants of the IDIs.

Participant C: When lying in bed, you cannot push yourself up; you cannot use your shoulders so you must laboriously roll in order to get on, on your knees next to your bed and then try to get your feet under yourself to get up. And then, well, try to sit on the toilet if, if you, if you don’t have any control.

(Man, IDI, aged 70–80 years)

Participant D: My husband, my husband had to carry [me] out of bed… I had to go to the toilet, he had to put me on the toilet. I had to take a shower, he had to shower me. Everything, because I couldn’t. I couldn’t even [hold] this glass in my hand because it just fell on the ground.

(Woman, IDI, aged 60–70 years)

Individuals reported other long-term daily life inconveniences, which could vary from crucial daily life activities to minor problems. Amongst these inconveniences where the inability to dress themselves because of impairment of movement, to wear shoes because of their swollen feet, to drive a car, to cook, to sport, to sleep well, and other inconveniences. It also affected the participants’ interpersonal relations with some mentioning that it was difficult for them to have sexual intercourse. A few of these were described as follows.

Participant E: I couldn’t dress myself (…) my husband had to help me. [If my husband would not be there to help] I would go without a bra [laughing] (…). Yes yes, I couldn’t do it [dressing], really, really.

(Woman, IDI, aged 50–60 years)

Participant B: eeeh. I couldn’t cook. That, that, that, heath, that heath of the kitchen was too much for me.

(Man, IDI, aged 30–40 years)

Participant D: Until today, from the seventh of October last year, I cannot wear high heels, I come to the office with slippers. Have to wear [them]. My ankles are entirely swollen.

(Woman, IDI, aged 60–70 years)

As a consequence of chronic chikungunya, people continued perceiving musculoskeletal impairment and other sequelae, leading to long-term dependency on others, loss of mobility and emotional concerns.

### Emotional impact of chikungunya

Within the community, different ways of how chikungunya influenced emotions were expressed. Depending on duration of disease and clinical presentation, people perceived minimal to major emotional impact caused by chikungunya. More specifically, individuals reported feelings of moodiness, anxiety, frustration, anger, and feeling left out, desperate, ashamed, confused or in some case perceived them self to be a different person. Emotional distress in the acute and chronic phase was related to lower knowledge on chikungunya. A distinction could be observed between emotions individuals perceived in the acute phase of disease and those referring to the long-lasting sequelae of chikungunya. Whereas initial emotional reactions were mainly triggered by the abrupt nature of the acute symptoms, the unpredictable and long-lasting nature of the chronic sequelae was responsible for an emotional impact on the longer term.

#### Emotions in the acute phase of chikungunya

Study participants perceived the acute phase of chikungunya as overwhelming. Anxiety was a commonly-described emotion and could be linked to lower reported knowledge of the symptoms and impact of chikungunya. This limited knowledge on chikungunya made it hard for participants to recognize associated complications, while participants thought they might be paralyzed, having a seizure, or even perceived dying from the disease.

Participant 1: I became nervous—Participant 2: Yes nervous—Participant 1: I asked the Lord, help me! Wat is happening here.–Participant 3: Yes even crying—FGD: Praying, ask God. Yes, because you were afraid.

(Women, FGD, aged 60–70 years)

Participant C: [I was afraid to fall asleep, because] I thought well, suppose that you eh just fall asleep and, and I can’t get it [hands/arms] started and that it just dies and that I then just *** [curse] have to miss a paw [hand/arm] because it died.

(Man, IDI, aged 70–80 years)

Within the community, it was believed that chikungunya might be lethal for people with a ‘weaker resistance [immune system]’ like little children, older people, or those with comorbidities. Consequently, family or friends of this ‘vulnerable’ group were in particular worried when these people got chikungunya.

Young men, normally relying on their physical strength explained that chikungunya was especially tough since they couldn’t rely on their bodies at that moment.

Participant B: But that, that, pain in my body wasn’t frustrating. So I can, I can easily handle that but I couldn’t do nothing at all and thát was frustrating. I could walk ‘aaah it hurts’, I still walked but ááh! The leg doesn’t work, nothing at all. And, thát was frustrating. But the pain was ‘ah yea, whatever’.

(Man, IDI, aged 30–40 years)

#### Emotions in the chronic phase of chikungunya

Participants had to live with pain, impairing the patient not only physically, but also emotionally. People perceived the long-lasting sequelae of chikungunya as unpredictable, which led to situations where they suspiciously evaluated symptoms, doubting whether their new or lingering symptoms were caused by chikungunya, or maybe by another disease. As participants were aware that there was no cure for chikungunya, this left them wondering if their pain and other symptoms would ever fade away. The perspective that the disease might possibly never be cured and that one might have to live with pain for a long time, left patients with feelings of hopelessness.

Participant F: That it hurts and you see that it, there’s no eeeh, no eeeh, there’s no way to remove the pain. There’s no eh and then you hear ‘o they [people] don’t have it [chikungunya] anymore?’ But I still have it! (laughs) you know? And then it stays and keeps lingering and lingering and that is depressing. You cannot do what you did earlier. For sure. (…) Oh! And then are there people who got it eh, only have it a week and then it’s completely gone. Nothing. (…) So that’s not fair. That’s depressing.

(Woman, IDI, aged 50–60 years)

Moreover, a perceived lack of efforts and resources to help chikungunya patients made them feel left out.

Participant G: Sometime the doctor tells you, ‘What now, what should we do now?’ and, ‘let’s try this and let’s try that’. Then you get the feeling that the person, the doctor himself doesn’t know what to do with ehm, with that chichingunya.

(Woman, IDI, aged 80–90 years)

The aforementioned feelings of helplessness were grounds for feelings of depression, which could be fostered when patients did not find themselves understood by others.

Participant 1: that was the thing the thing that people kept stating: it is not curable and there is no treatment. With the result that you sat at home and kept fretting about what you have and that there is no way to cure.

Participant 2: You go outside to do what? To talk with people? And then? What are you going to talk about? Because, they are not going to help you. And you start thinking nobody can never help me.

(Women, FGD, aged under 30 years)

Besides that, the emotional impact that was caused by hopelessness and helplessness resulting from ongoing chronic sequelae bothered chikungunya patients in their daily lives. Patients narrated that the unceasing pains made them easily irritable or temperamental during the chronic phase of chikungunya.

Participant H: I, I just didn’t know that I would become such a *** [curse for someone complaining a lot] you know? You can really turn temperamental by it [by the situation of having chronic chikungunya].

(Woman, IDI, aged 40–50 years)

This moodiness was also perceived by the people surrounding chikungunya patients, which was reflected when unaffected people in FGDs spoke about chikungunya patients, as follows:

FGD: You hear people giving comments. Being mad. They talk dirty words.–Participant 1: I think I’ve heart from people that you get a bit irritated, you know? Because you remain with pain continuously.–Participant 2: It works on your nerves. It is like [name] also said, you start fights more easily. You become mad.

(Men and women, FGD, aged 50–80 years)

A significant topic on emotional impact emerging from FGDs and IDIs was the perceived accelerated aging of the body. This perceived aging was mainly linked to the pain and physical impairment, which is a frequent sign of aging. However, the sudden onset of aging forced patients to adapt their lifestyle, which was reported to make people feel like having transformed into another person by chikungunya.

Participant H: I feel like 80. (…) But I want to be again… what I want is just don’t feel anything anymore, that I am again like … come on, you know, I used to do classic ballet? I mean, I am just [in my young forties]. Then it should be possible to become the old [me] again? That’s it.

(Woman, IDI, aged 40–50 years)

Commonly, participants stated that chikungunya had turned them into another person due to the perceived aging of their body, to moodiness or due to loss of vitality. Moreover, some participants perceived having turned into another person because they were unable to do things, which were part of their identity, like dancing, wearing high heels, going out or cooking.

Participant G: I love physical exercise, I love to go out I love, eh having fun. I love eh.. eh.. cooking. I love everything what can keep me busy, that’s what I love. But when I got that chichingunya, it was, it was gone …. [long silence, woman starts crying] …. Sorry (…) I had never thought that a disease can transform a person like this.

(Woman, IDI, aged 80–90 years)

Some older adults became (more) dependent on care of others. For them, the question of how long chikungunya would last became a significant question, since independency was an important value for these people. The fact that their physical condition had prompted their family or friends to care for them was difficult to accept. Some of the older adults narrated how during intense disease manifestations, they were more willing to accept the possibility of death. Older adults having severe chronic complaints were (more) dependent on care of others.

Participant I: So like I told you before, I have always asked God that I don’t want to be a burden to anyone. So, if the day comes that I cannot do anything anymore, God takes me.

(Man, IDI, aged 70–80 years)

The emotional consequences of chikungunya varied, and in some cases left people with uncertainty about their future and indecisiveness on what to do next.

### Impact of chikungunya on social life

Among the community, chikungunya was held responsible for varying consequences regarding social life of patients. These consequences varied depending on manifestation, duration and severity of disease and concerned inabilities to join social activities or to visit friends and being avoided by family or friends. However, some patients had not experienced any impact on their social life. This could be the case when people did not have a social life of notice, or if disease manifestations were so mild that no impact on social activities was perceived.

Individuals narrated how they, due to their physical impairments and loss of vitality, could not join social activities such as going to the cinema, compete in sport matches, drinking with friends, play golf, do physical exercise or walk with friends. These created a situation where participants could perceive weakening of social bounds.

Participant B: Normally yes, I went out, I go to movies or eh, (…) Do a drink with friends, yes…. But back then with the chikungunya I just did nothing. Just stayed at home, calm.

(Man, IDI, aged 30–40 years)

Participant F: Yes I eh at Thursdays, every Thursday I walk with a group into the mondi [forest, country side] and I have not done that for months because I cannot bend and crawl because it hurts.

(Woman, IDI, aged 50–60 years)

Others explained that even if the activity only concerned visiting friends, they would not be able to go, because they were not able to drive a car, due to the symptoms of chikungunya. Furthermore, people stated not being in the mood of acting social, as was also reflected before in the section ‘emotional impact of chikungunya’.

Limited knowledge on transmission routes of disease could cause an impact on social life. Namely the belief that people with chikungunya could infect others left some participants without having their family or friends visiting them. Also, some participants did not want to visit others, since they were afraid to infect them.

Participant J: Yes but you know that if you sometimes stay to talk [with people] you could infect them so I rather stayed at home.

(Woman, IDI, aged 50–60 years)

The belief that chikungunya was contagious by patient contact was not the only reason to avoid visiting others. Participants described how family living abroad cancelled their planned stay, or did not come because they were afraid of getting chikungunya.

Participant I: I have a brother who didn’t want to come. (…) Because of chikungunya. He already has arthritis problems and he wants to prevent additional [articular problems]. He looks to me and says ‘no thank you!’ He didn’t go to a funeral of my mother. And afterwards he hasn’t come either because of chikungunya.

(Woman, IDI, aged 50–60 years)

Thus, social impact of chikungunya was not perceived as substantial, but could still lead to social isolation.

### Coping with the impact of chikungunya

Participants reacted to the consequences of disease described above by performing different coping strategies, which we describe in the following sections.

Within the community, problem-focused coping was commonly performed by using (natural) medicines and by visiting a physician to cope with the physical impact of chikungunya. In general, chikungunya patients stayed in bed during the acute phase of chikungunya and depended on the care of family or friends. Their physical condition permitting, most of them went to see a physician, normally accompanied by someone to transfer them. Otherwise, a physician was visited one or two days later.

Some of the participants did not visit a physician. These participants did not recognize visiting a physician as a problem-focused coping strategy, because they would not be treated with more than the regular symptom relief-providing medications, i.e. paracetamol and nonsteroidal anti-inflammatory drugs. Furthermore, some participants found the symptoms obvious and consequently did not want to waste time to seek the opinion of a physician. At some point of time during the epidemic, participants reported that people were not being laboratory tested any more, and this also discouraged people from visiting a physician. Although some decided not to go to see a physician, others decided to visit a physician many times (up to approximately 20 times in some cases) for the complaints caused by chikungunya. Frequent physician visits were linked to desperation, a desire for solace, persistence of symptoms, and a desire to try new treatment. Frequent physician visits were also performed because people needed to prove disease persistence in order to be reimbursed for the absence from their work.

Participant D: Every week I was at the doctor, because I had pain here, pain at the feet, uh! (…) it didn’t disappear and according to me I say ‘yes, there should be something that I can use or so’ (…) I don’t know if 20 times, the first months for sure every day at the doctor told me ‘I can really not do anything for you’.

(Woman, IDI, aged 60–70 years)

Many participants had used medicines to treat their physical complaints. Those included the medicines bought over the counter or prescribed by the physician, amongst which were mainly paracetamol or NSAID’s. Furthermore, natural or other alternative medicines were very popular since they had been prominently presented in advertisements and media by alternative healers. Hence, people recommended these medicines to each other. Some individuals with persistent complaints reported to have used more than ten different medicines. Amongst the most popular medicines were mango and papaya leaves, lemon grass, aloe and vitamin B.

Participant E: Everyone, so many people got it [chikungunya]. Because all say ‘do you know what I took? Do you know what I have done?’ So you hear from everybody and yes, you try [the promoted medicines].

(Woman, IDI, aged 50–60 years)

Using frequent physician visits and different medical treatments was a pattern of problem-focused coping, which was illustrative for people who perceived severe impairment from chikungunya, which they could hardly accept. Frequent visits of health care providers as coping strategy led to unmet expectations for cure or treatment, resulting in stress (negative impact on QoL). In contrast, people with lingering complaints while reporting less impact of chikungunya also showed different coping strategies, i.e. emotional coping strategies. The participants using emotional coping strategies went less often to a physician, used less medications and reported trying to do as many physical/social activities as possible. For example, they tried to mobilize by performing the physical exercises or movements they could do. With this in mind, people were more willing to accept that symptoms would eventually subside.

Participant C: maybe it is my mindset too, I mean see it a bit philosophic. I mean it goes… it’s the truth of matter and it goes… and if you worry about it you will perceive a higher burden so eh, you know it when you see it.

(Man, IDI, aged 70–80 years)

Participants believed that chikungunya stayed in the system (the body), which made them susceptible for lingering symptoms during that time. This period of having ‘chikungunya in the system’ could range from 6 months up to 2 years. During the chikungunya epidemic, people believed that a ‘high resistance’ [immune system] was a vital way of preventing chikungunya, or to make sure that chikungunya would not cause major symptoms or linger on during infection. Some participants combined the latter two perceptions, and consequently coped with chronic chikungunya sequelae by living healthy during the time that they thought that chikungunya would be in their system, in order to diminish the lingering of chikungunya symptoms.

Participant: it is not to eat healthy, the vitamins etcetera, not to eat healthy but the resistance [against diseases] should stay high, so that it [chikungunya symptoms] doesn’t return.

(Woman, FGD, aged 60–70 years)

Actions participants performed to ‘strengthen the immune system’ were: eating healthy, physical activity, not visiting sex workers and using herbs or vitamins.

Individuals coped with social consequences by calling their family and friends instead of visiting them. Social HRQoL impact was also diminished by emotional coping strategies relying on social support. People passed by to inform about the situation of the ill people and tried to help each other by offering advices and remedies, or made fun of each other. Moreover, people started helping each other by bringing people to the physician or to the Social Insurance Bank. Others obtained emotional support from others in order to deal emotionally with chikungunya sequelae.

Participant: The community, (…) not specifically that is cures, but that it makes you feel better because someone understood what’s going on.

(Woman, FGD, aged under 30 years)

The financial impact due to chikungunya on participant’s personal situation was generally perceived as limited. In Curaçao, chikungunya patients could rely on an insurance system, which covered health care costs, which in turn minimized out-of-pocket expenditures. These insurances covered also parts of the income loss of chikungunya patients who could not work. However, participants pointed to financial impact on society level. This was attributed to the fact that chikungunya had left many people at home instead of going to their work, and because the epidemic might have prevented tourist from visiting Curaçao.

## Discussion

Chikungunya constitutes an emerging public health problem, mainly due to its debilitating acute and long-term musculoskeletal manifestations. Though the disease can cause major impairment of Quality of Life (QoL), this is the first study conducted to obtain an in-depth understanding of the impact of chikungunya on Health Related (HR)QoL of its patients using qualitative research methods. HRQoL of participants were mainly affected on physical and emotional domains, and to a lesser extent on social and financial domains. Problem-focused coping strategies aimed at physical healing induced a high uptake of (alternative) medicines and consultation of physicians, whereas emotional coping strategies focused on acceptance of the situation were linked to lower perceived impacts on HRQoL and less uptake of medical care.

Chikungunya patients reported an acute and chronic disease presentation in agreement with the existing literature [30–35]. Additionally, a prodromal period before disease onset and acute symptoms like ‘a change in sense of taste’ and incontinence were reported. The latter findings are interesting but should be cautiously interpreted since participant expressed their confusion regarding chikungunya sequelae, and no comprehensive clinical assessment of the participants of this study was performed. This confusion and wide variety of symptoms that participants linked to acute and chronic chikungunya are in agreement with the current situation of knowledge on chikungunya. Clinical studies are showing a wide variety of disease manifestations [10], yet an all-encompassing consensus on the clinical picture of chikungunya is lacking.

The acute and chronic disease presentation of chikungunya was directly responsible for impairment of physical HRQoL, and demonstrated indirect consequences for emotional HRQoL. The acute disease was described as highly debilitating in which patients perceived strong anxious emotions, sometimes to the extent that they thought they might be dying. This could have partly been prevented if people had been timely informed of the sudden and tough situation they might face when acquiring chikungunya. Providing timely information is important and a key to reduce health-related distress [36,37]. During chikungunya outbreaks, it is important that health authorities proactively provide messages on the symptoms and normally non-lethal nature of chikungunya.

The unpredictable lingering nature of mainly musculoskeletal manifestations and its subsequent difficulties in normal daily life activities were linked to several consequences for emotional HRQoL. Patients narrated how these sequelae influenced their mood and ability to perform their daily-life activities. Hence, chikungunya had turned participants into ‘other persons’. Young men linked their physical strength to a perceived resistance to diseases, as was also described in a study in Cambodia [38]. When these young men were hit by chikungunya and perceived long-lasting impairment of their physical strength, loss of part of their identity caused emotional bearing. The same was observed when women could not wear high heels, dance or go out, thus impacting negatively on their identity. The notion that chikungunya affected patients’ identity was also reflected in the perceived accelerated aging of the body. Being independent is an important value for elderly [39,40]. Therefore, by rendering elderly individuals dependent of care, chikungunya could have major emotional consequences for these people. Knowledge of the aforementioned emotional concerns is valuable to all health professionals directly working with chikungunya patients, and advocate for the need to target emotional concerns in health promotion policies.

Health related impact on social life and the personal financial situation of participants were limited, since ways of coping diminished the significance of these concerns. The universal health care program (via the Social Insurance Bank or other) covered generally the health care expenses. In the countries where people cannot rely on a universal health insurance scheme such as the one available in Curaçao, it is expected that chikungunya patients will face a higher financial burden. Consequently, chikungunya has been shown to cause major financial burdens on society level across Latin America [41].

The perception that chikungunya is transmissible from patient to patient should be targeted in health campaigns by promoting transmission methods. Improved knowledge on transmission routes of chikungunya could hereby take away the barrier to engage in social activities when having chikungunya. Enhancing social activities may additionally improve coping with the impairment of emotional HRQoL [36], as social support is an important mediator between life stress and health status [24].

This study describes several ways of how participants coped with the effects of chikungunya. A notable coping strategy was high uptake of (natural) medicines or frequent physician visits, which was typically performed by subjects with major impacts on HRQoL and difficulties to accept their situation. Since effective long-term treatments are not available yet for chronic chikungunya, the latter coping strategy will normally not lead to success. The consequent dissatisfaction may only give rise to further emotional stress [42,43]. Hence, health professionals should identify patients performing this coping strategy to promote healthier coping strategies. For example, participants showing coping strategies focused on acceptation of circumstances or on social support showed better HRQoL, which is in line with literature on coping strategies [24,36]. Some people solely relied on (natural) medicine or alternative healers; instead of attending physicians, as it was also reported from other febrile diseases and populations [e.g. 44,45]. Promotion of healthy coping strategies amongst these people might be a challenge, but may be achieved via media or community centres.

Another coping strategy included a ‘healthy lifestyle as long as chikungunya is in the system’. ‘Living healthy’ has also been described as a coping strategy with a possible dengue infection [38]. Currently, there is no scientific substantiation for this coping strategy. Nevertheless, because no effective long-term treatment for chikungunya sequelae is present, this way of coping will under normal conditions only improve health.

This study used different qualitative methods. Since the participants of the IDIs (except for the family members of chikungunya patients) had their chikungunya infection serological confirmed, we focused our analysis mainly on the laboratory-confirmed chikungunya patients of the IDIs. The FGDs were used to understand perceptions of the community.

This study was limited by its qualitative study design. As is normal for qualitative research, participants were not randomly selected. Therefore, generalization of these findings is limited to this specific study site and study population. Furthermore, HRQoL is a broad concept. Although this study was explorative and comprehensive in nature, it might not have captured all aspects of the HRQoL. A researcher bias may be present in this study. We minimized this by involving the local research group and the research group in the Netherlands in the different stages of the study (i.e. study methods, data interpretation and data presentation). Additionally, a contamination bias due to sharing of chikungunya knowledge and study procedures between investigated communities might be present. We assume that this was no major issue, since this study mainly focused on personal experiences of chikungunya episodes. Furthermore, the issue of chikungunya was already widely discussed within Curaçao both in the community and in the media.

Because it is vital to understand the impact of a disease in order to develop applicable and effective health intervention, these interventions should be grounded in research on HRQoL. This study is the first applying qualitative research methods on this topic and providing valuable new in-depth insights on HRQoL and coping strategies concerning acute and chronic chikungunya sequelae. These insights help health professionals to understand the impact of chikungunya on its patients. The results reveal that health promotions strategies could be improved, by (1) providing timely messages to the public about the devastating but normally non-lethal nature of chikungunya, to ease chikungunya patients during acute disease, (2a) promoting emotional coping strategies and (b) targeting ‘unhealthy’ problem-focused coping strategies among chronically affected chikungunya patients, (3) focusing on providing (emotional) support to older chikungunya patients.

Further research could include health professionals working in chikungunya endemic areas. This would allow to assess clinical management (consultation, treatment recommendations) of chikungunya, and to provide insight on how this was put in practice by the patients. Similar qualitative studies should be performed in different settings, as these may serve as basis for randomized surveys on HRQoL and coping strategies of chikungunya.

## Supporting information

S1 Table COREQ—Consolidated criteria for reporting qualitative studies. (PDF)

S1 Text In-depth interview guide. (PDF)

S2 Text Focus group guide. (PDF)
