# Supplementary material for: Health-related impact on quality of life and coping strategies for chikungunya: A qualitative study in Curaçao
Source: PLoS Negl Trop Dis. 2017 Oct 9;11(10):e0005987. doi: 10.1371/journal.pntd.0005987 (PMC5648258; doi:10.1371/journal.pntd.0005987)
Supplement: S1 Table — (PDF) [file pntd.0005987.s001.pdf]

Consolidated criteria for reporting qualitative studies (COREQ): 32-item checklist

No	Item	Guide questions/description	Answer-/ position in article
**Domain 1: Research team and reflexivity**			
Personal Characteristics			
1.	Interviewer/facilitator	Which author/s conducted the interview or focus group?	See Methods – Data Collection
2.	Credentials	What were the researcher's credentials? *E.g. PhD, MD*	See box on the left.
3.	Occupation	What was their occupation at the time of the study?	
4.	Gender	Was the researcher male or female?	
5.	Experience and training	What experience or training did the researcher have?	
**Answers on criteria 2-5**.<br>Authors:<br>- Jelte Elsinga, BSc – male, MD PhD candidate, master courses in qualitative research methods.<br>- Martin P. Grobusch MD PhD – male, professor, extensive experience in clinical practice, research and public health.<br>- Adriana Tami MD PhD – female, assistant professor, extensive experience in clinical research and public health.<br>- Izzy Gerstenbluth MD – head of epidemiology and research unit Curaçao, extensive experience in clinical practice and public health of Curaçao.<br>- Ajay Bailey PhD – male, assistant professor, anthropologist with extensive experience in qualitative research and health behaviour.<br><br>Interviewers:<br>- Martha Gillen – female, retired public health nurse.<br>- Billy Hoogenbergen – male, retired psychotherapist.			
Relationship with participants			
6.	Relationship established	Was a relationship established prior to study commencement?	No
7.	Participant knowledge of the interviewer	What did the participants know about the researcher? e.*g. personal goals, reasons for doing the research*	There was an informed consent taken, signed by all participants.
8.	Interviewer characteristics	What characteristics were reported about the interviewer/facilitator? e.g. *Bias, assumptions, reasons and interests in the research topic*	Researcher-bias; see Discussion.
**Domain 2: study design**			
Theoretical framework			
9.	Methodological orientation and Theory	What methodological orientation was stated to underpin the study? *e.g. grounded theory, discourse analysis, ethnography, phenomenology, content analysis*	Principles of grounded theory for analyses, but theory informed empirical fieldwork. See methods - Data collection
Participant selection			
10.	Sampling	How were participants selected? *e.g. purposive, convenience, consecutive, snowball*	See methods - Study design and participants recruitment
11.	Method of approach	How were participants approached? e.*g. face-to-face, telephone, mail, email*	See methods - Study design and participants recruitment
12.	Sample size	How many participants were in the study?	See Table 1
13.	Non-participation	How many people refused to participate or dropped out? Reasons?	Refused: not known for those recruited via physicians; Dropped out: none.
Setting			
14.	Setting of data collection	Where was the data collected? e.*g. home, clinic, workplace*	See Methods – Data collection
15.	Presence of non-participants	Was anyone else present besides the participants and researchers?	See Methods – Data collection
16.	Description of sample	What are the important characteristics of the sample? *e.g. demographic data, date*	See Table 1
Data collection			

17.	Interview guide	Were questions, prompts, guides provided by the authors? Was it pilot tested?	Yes. See Methods – Data collection & Supporting Information 1 and 2
18.	Repeat interviews	Were repeat interviews carried out? If yes, how many?	No
19.	Audio/visual recording	Did the research use audio or visual recording to collect the data?	Yes. See Methods – Data collection
20.	Field notes	Were field notes made during and/or after the interview or focus group?	Yes. See Methods – Data collection
21.	Duration	What was the duration of the interviews or focus group?	In-depth interviews: 35-100 minutes<br>Focus group discussions: 65-85 minutes
22.	Data saturation	Was data saturation discussed?	Yes. See Methods – Data collection
23.	Transcripts returned	Were transcripts returned to participants for comment and/or correction?	No
**Domain 3: analysis and findings**			
Data analysis			
24.	Number of data coders	How many data coders coded the data?	1
25.	Description of the coding tree	Did authors provide a description of the coding tree?	See Methods – Data Analysis
26.	Derivation of themes	Were themes identified in advance or derived from the data?	See Methods – Data Analysis
27.	Software	What software, if applicable, was used to manage the data?	See Methods – Data Analysis
28.	Participant checking	Did participants provide feedback on the findings?	No, but findings were discussed with local experts.
Reporting			
29.	Quotations presented	Were participant quotations presented to illustrate the themes / findings? Was each quotation identified? e.*g. participant number*	See Results
30.	Data and findings consistent	Was there consistency between the data presented and the findings?	See Results
31.	Clarity of major themes	Were major themes clearly presented in the findings?	See Results
32.	Clarity of minor themes	Is there a description of diverse cases or discussion of minor themes?	See Results
