# Supplementary material for: Health-related impact on quality of life and coping strategies for chikungunya: A qualitative study in Curaçao
Source: PLoS Negl Trop Dis. 2017 Oct 9;11(10):e0005987. doi: 10.1371/journal.pntd.0005987 (PMC5648258; doi:10.1371/journal.pntd.0005987)
Supplement: S1 Text — (PDF) [file pntd.0005987.s002.pdf]

Interview guide – in-depth interviews QoL Chikungunya

This interview guide was designed and used in Dutch and Papiamentu, and upon publication of the study translated to English. This is a summarized version of the original interview guide. Only the parts that were relevant for this study were translated and presented.

Introduction

This concerned a repetition of the purposes of this study, the interview procedures, ethical considerations and the rights of the interviewer. After this, the participant had an additional chance to ask questions.

Background information

Sex, place of residence, living situation (with whom?), education, employment, country of origin participant's parents, country of origin of participant, religion.

A. Opening questions

1. When is somebody ill?

Probe: severity, symptoms, daily life limitations

2. What should somebody do when he/she has a fever?

Probe: wait (how long?), treat at home (with what?), doctor, traditional treatment

3. Could you describe the most common diseases in Curaçao?

Probe: short-lasting diseases, long-lasting diseases, dengue, chikungunya

B. Knowledge about chikungunya

1. Via which sources/where have you heard about chikungunya?

Probe: mass media (television, newspaper, internet), government, family/friend/acquaintances, medical centres

2. How does a person get chikungunya?

Probe: mosquito, air, being near to a chikungunya patient, water
2b. How did you get chikungunya?

3. Can someone protect him/herself against chikungunya? How?

Probe: Deet, Aloe, long-sleeved clothes, van/air conditioning
3b. How do you protect yourself?
3c. How did you protect yourself (before getting chikungunya) against chikungunya?

4. What are the symptoms of chikungunya?

Probe: Fever, joint pain, skin diseases, hair loss, muscle aches

C. Risk perception

1. What put you at risk for getting chikungunya?

Probe: places, activities, role of preventive measures

The next question concerns the time before you got chikungunya. I would like to ask you to remember as good as possible your situation at that time.

2. Had you expected to get chikungunya? Why/ why not?

D. Attitudes and choices during chikungunya infection

The following questions concern the disease course of your chikungunya infection. When answering these questions, try to remember as good as possible what choices you made at the time of disease.

Interview guide – in-depth interviews QoL Chikungunya

It could be possible that at this time, you would have done something else. Therefore, you will get the opportunity at the end of the interview to reflect on this. But now, I would like to know why you made certain choices at the time you had chikungunya.

1. How long ago started the symptoms of chikungunya?

2. What were your first thoughts about the cause of the symptoms? Why?

Probe: dengue

3. What made you realize that you had chikungunya?

Probe: fever, symptoms

4. What was the first thing you did when you knew you were ill? Why?

Probe: reason for this behaviour, desired effect, how long and when, evaluation (did it work?)

5. What was the next thing what you did? Why?

Probe: reason for this behaviour, desired effect, how long and when, evaluation (did it work?), next action

6. What medication did you use?

Probe: why (+inducement), recommendation (by whom?), when, how much

7. Could you describe what symptoms you had and how long they lasted?

8. Which caregivers did you consult?

Probe: general practitioner, hospital, traditional healer, why (+inducement), when, evaluation (did it work?)

E. Barriers in health seeking behaviour

1. Did you face difficulties to obtain the care you desired? If yes: Which?

Probe: Fast diagnosis, access to- and availability of care, quality of care, costs, daily life duties

F. Impact of the chikungunya infection

1. How severe is chikungunya? Why?

Probe: lethal, hospital-admission, doctor visit, social/economic consequences

2. What impact has the chikungunya infection had on you?

Probe: physical, emotional, financial, social (family/friends)

3. Do you experience the consequences of chikungunya disease, at this moment? What consequences/how?

Probe: physical, emotional, financial (work), social

4. How severe is a dengue infection? Why?

Probe: Compared to chikungunya.

G. Evaluation of disease course and health seeking behaviour

1. Looking back to what you did when you had chikungunya: would you have done something different if you got chikungunya now?

Probe: go earlier to the doctor, don’t go to the doctor, wait longer, medication, rest

2. What would you advise to people with chikungunya (what should they do?)?

Probe: treatment at home, doctor, medicines, herbs

3. In your opinion, what could the government do to help people with a chikungunya infection?

Probe: Health seeking behaviour themes.

H. Would you like to say anything else?
