# Supplementary material for: Health-related impact on quality of life and coping strategies for chikungunya: A qualitative study in Curaçao
Source: PLoS Negl Trop Dis. 2017 Oct 9;11(10):e0005987. doi: 10.1371/journal.pntd.0005987 (PMC5648258; doi:10.1371/journal.pntd.0005987)
Supplement: S2 Text — (PDF) [file pntd.0005987.s003.pdf]

Focus group guide – Focus group discussions QoL Chikungunya

This interview guide was designed and used in Dutch and Papiamentu, and upon publication of the study translated to English. This is a summarized version of the original interview guide. Only the parts that were relevant for this study were translated and presented.

Introduction
This concerned a repetition of the purposes of this study, the interview procedures, ethical considerations and the rights of the interviewer. After this, participants had an additional chance to ask questions. Then, participants shortly introduced themselves.

Opening questions
- Could you describe the health problems in Curaçao?
- What is your opinion about the quality of the health care in Curaçao?

Topic 1 Knowledge about chikungunya

1. Where do people obtain information from about chikungunya?
   Probe: what kind of information?
2. What do you know about chikungunya?
   Probe: cause of disease: mosquito, symptoms: acute & long-lasting, protection against chikungunya

Topic 2 Health seeking behaviour of chikungunya

3. Who can get chikungunya? What puts people at risk for getting chikungunya?
   Probe: Men/women, places, activities
4. How do people on Curaçao protect themselves against chikungunya?
   Probe: DEET, long-sleeved clothes, removal of mosquito breeding sites
5. What do people on Curaçao do when they get chikungunya? Why?
   Probe: treat chikungunya at home (with *herbs*), medication, doctor (where), traditional healer.
6. Some people said that they use(d)/tried many different medicines for chikungunya. What do you think about that?
7. Some people visited a doctor more than 10 times for chikungunya, what are reasons for this? What is your opinion about this?
   Probe: People are desperate?

Topic 3 The impact of chikungunya

8. What are the physical consequences of a chikungunya infection?
   Probe: short term, long term
9. What are the social consequences of a chikungunya infection?
   Probe: contact with family/friends, performance of normal daily life activities
10. What is the emotional impact of a chikungunya infection?
    Probe: depression, temperamental/moodiness
11. What financial costs does a chikungunya patient have?
    Probe: medication, costs of treatment, loss of income, short and long term costs
12. What is the impact on the family/friends who care for the chikungunya patients?
13. Do people die because of chikungunya?
14. One of the participants told: ‘Chikungunya can stay for two years in your system, so you must be strong during this time (/have a good immune system), because chikungunya strikes when you are weak, on your weak spots.’
    What is your opinion about this?

Topic 4 Recommendations towards policy of chikungunya

15. What can be done to improve the help/care of chikungunya patients?

Probe: Aid of patients with long-lasting complaints, role of government, role of doctors.

Conclusion
Do you have further comments on, or additions to this discussion?
